# Magnetic Mesoporous Silica for Targeted Drug Delivery of Chloroquine: Synthesis, Characterization, and In Vitro Evaluation

**DOI:** 10.3390/pharmaceutics16030357

**Published:** 2024-03-03

**Authors:** Rafaela de Andrade, Rita de Cássia dos Reis Schmidt, Leonardo Santos Gomes, Legna Colina-Vegas, Ruth Hinrichs, Marcos Antônio Zen Vasconcellos, Tania Maria Haas Costa, Monique Deon, Wilmer Villarreal, Edilson Valmir Benvenutti

**Affiliations:** 1 Instituto de Química, Universidade Federal do Rio Grande do Sul (UFRGS), Porto Alegre 91501-970, RS, Brazillegna.vegas@ufrgs.br (L.C.-V.); wilmer.villarreal@ufrgs.br (W.V.); 2 Programa de Pós-Graduação em Biociências, Universidade Federal de Ciências da Saúde de Porto Alegre (UFCSPA), Porto Alegre 90050-170, RS, Brazil; 3 Instituto de Geociências, Universidade Federal do Rio Grande do Sul (UFRGS), Porto Alegre 91501-970, RS, Brazil; ruth.hinrichs@ufrgs.br; 4 Instituto de Física, Universidade Federal do Rio Grande do Sul (UFRGS), Porto Alegre 91501-970, RS, Brazil; marcos@if.ufrgs.br

**Keywords:** antimalarial, chloroquine, enhanced drug solubility, MCM-41, magnetic nanocarriers

## Abstract

Malaria is a dangerous tropical disease, with high morbidity in developing countries. The responsible parasite has developed resistance to the existing drugs; therefore, new drug delivery systems are being studied to increase efficacy by targeting hemozoin, a parasite paramagnetic metabolite. Herein, magnetic mesoporous silica (magMCM) was synthesized using iron oxide particles dispersed in the silica structure for magnetically driven behavior. The X-ray diffractogram (XRD) and Mössbauer spectra show patterns corresponding to magnetite and maghemite. Furthermore, Mössbauer spectroscopy revealed superparamagnetic behavior, attributed to single magnetic domains in particles smaller than 10 nm. Even in the presence of iron oxide particles, the hexagonal structure of MCM is clearly identified in XRD (low-angle region) and the channels are visible in TEM images. The drug chloroquine (CQ) was encapsulated by incipient wetness impregnation (magMCM-CQ). The N_2_ adsorption–desorption isotherms show that CQ molecules were encapsulated in the pores, without completely filling the mesopores. BET surface area values were 630 m^2^ g^−1^ (magMCM) and 467 m^2^ g^−1^ (magMCM-CQ). Encapsulated CQ exhibited rapid delivery (99% in 3 h) in buffer medium and improved solubility compared to the non-encapsulated drug, attributed to CQ encapsulation in amorphous form. The biocompatibility assessment of magMCM, magMCM-CQ, and CQ against MRC5 non-tumoral lung fibroblasts using the MTT assay after 24 h revealed no toxicity associated with magMCM. On the other hand, the non-encapsulated CQ and magMCM-CQ exhibited comparable dose–response activity, indicating a similar cytotoxic effect.

## 1. Introduction

Malaria is a tropical disease caused by the protozoan *Plasmodium* sp. and it can be fatal. According to the 2022 World Health Organization (WHO) malaria report, the estimative of malaria cases in 2021 were 247 million cases, 2% of which ended in death. The disease occurs mainly in tropical areas of developing countries. For example, Venezuela, Brazil, and Colombia together accounted for more than 79% of the cases on the American continent [1,2].

Unfortunately, *Plasmodium*’s resistance to existing drugs has increased over the years. The investigation of the reasons for this increase indicates that the resistance mechanism is associated with protein polymorphism, which alters *Plasmodium*’s physiological regulation, which is caused by genetic factors [3,4]. When the protozoan becomes resistant, a greater drug concentration is needed to combat it, which can increase the adverse events in patients. For example, chloroquine (a common antimalarial drug) causes side effects on several body tissues when increased doses are used [5,6].

To overcome these problems, new drug research [7,8] and drug delivery systems are being studied. Encapsulated drugs present an improvement in pharmacokinetics and efficiency when compared to free drugs. They can also mitigate unwanted features, such as low solubility, high toxicity, and untargeted delivery [9]. Therefore, encapsulated malaria drugs can be an alternative to decrease *Plasmodium*’s resistance to drugs already widely used in endemic regions, because these systems can offer the possibility of the same drugs being used in lower concentrations and with targeted delivery [10,11]. Diverse systems to encapsulate antimalaria drugs have been studied, such as metallic nanoparticles [12,13], lipid nanoparticles [14], polymers [15], hydrogels [16], and mesoporous silica [17,18].

Silica-based materials are a good option to encapsulate drugs due to their well-known characteristics of thermal, mechanical, and chemical stability, as well as biocompatibility. Among the mesoporous silica, MCM-41 (Mobil Composition of Matter) has hexagonally ordered pore channels, high surface area, and tunable pore size. Therefore, MCM-41 can be a drug nanocarrier with the additional capacity of enhancing the solubility of hydrophobic drugs. The enhancement is due to the prevalence of the drug’s amorphous form when encapsulated, which increases the drug dissolution/release rate and bioavailability. This feature is provided by the pore confinement effect, which can be achieved using MCM-41 as a nanocarrier [19,20,21].

During *Plasmodium*’s development inside the erythrocytes (red blood cells), the parasite detoxifies itself through polymerization of the heme group mechanism, which forms hemozoin, also known as malarial pigment. This pigment has paramagnetic behavior, which under the effect of strong magnetic fields can attract magnetic materials [22]. Due to this, antimalarial drugs encapsulated in magnetic materials can be an advantage in targeted therapies. However, there are few published studies about magnetic drug delivery systems applied to malaria treatment [23,24]. In this context, magnetic iron oxide nanoparticles stand out in medical applications due to their stability and biocompatibility. Iron oxide nanoparticles are commercially used (FDA-approved) as contrast agents in nuclear magnetic resonance (NMR) imaging and for cancer and anemia treatments [25,26,27].

Therefore, in this work, a magnetic MCM-41 was synthesized with adequate mesoporous structure and appreciable magnetization. The magnetic, crystallographic, morphologic, textural, and thermal properties of the materials were obtained and the magnetic MCM loaded with the antimalarial drug chloroquine was tested in vitro as a drug delivery system. An MTT assay was conducted to evaluate the biocompatibility of the materials.

## 2. Materials and Methods

### 2.1. Chemicals

Hexahydrate ferric chloride (Vetec, Duque de Caxias, RJ, Brazil), trihydrate sodium acetate (Dinâmica, Indaiatuba, SP, Brazil), ethylene glycol (Dinâmica, Indaiatuba, SP, Brazil), cetyltrimethylammonium bromide (CTAB, Vetec, Duque de Caxias, RJ, Brazil), tetraethyl orthosilicate (TEOS, 98%, Sigma Aldrich, Saint Louis, MO, USA), ammonium hydroxide (25%, Merck, Darmstadt, Germany), and methanol (Merck, Darmstadt, Germany) were used as purchased. Chloroquine was obtained after the reaction of chloroquine diphosphate (Sigma Aldrich, Saint Louis, MO, USA) with ammonium hydroxide (Nuclear, Diadema, SP, Brazil) in chloroform (Neon, Suzano, SP, Brazil) as solvent. Aqueous solutions were prepared in distilled water.

### 2.2. Synthesis of CQ-Loaded Magnetic Mesoporous Silica

The synthesis of the magnetic phase was carried out using the solvothermal method. At first, hexahydrate ferric chloride (2.16 g, 8 mmol) was dissolved in ethylene glycol (100 mL) under magnetic stirring. Then, trihydrate sodium acetate (4.8 g, 35 mmol) was added. After 1 h of magnetic stirring, the resulting solution was transferred to an autoclave with a Teflon inner container and heated to 180 °C at a heating rate of 3 °C min^−1^. After 6 h the system was cooled to room temperature, and the black magnetic precipitate was separated using a magnet, washed several times with ethanol, and dried under vacuum for 2 h. The sample was named starting iron oxide.

The synthesis of mesoporous silica containing iron oxide was based on previously reported syntheses for MCM-41 silica [28,29]. The surfactant CTAB (1.2 g, 3.3 mmol) was dissolved in water (120 mL) at 50 °C. Then, ammonium hydroxide (4.5 mL) was added as a catalyst and the starting iron oxide magnetic material (0.75 g) was mixed under mechanical stirring. TEOS (5.7 mL, 25.5 mmol) was added dropwise, and the reaction was maintained under stirring at room temperature for 24 h. Afterward, the reaction mixture was transferred to an autoclave and kept at 100 °C for an additional 24 h. Subsequently, the material was separated magnetically, washed with water, and dried in an oven at 80 °C for 4 h. Then, it was calcined at 550 °C for 6 h to remove the surfactant. The resulting solid was named magMCM.

To encapsulate the CQ drug in magMCM, the incipient wetness method was employed. A solution of CQ in methanol (47 mmol L^−1^) was slowly dripped onto previously dried magMCM-41 (1 h at 90 °C) until paste point formation. Subsequently, the material was left at room temperature for 2 days for solvent evaporation. The resulting solid was named magMCM-CQ. The drug loading percentage was defined as the amount of CQ in 100 mg of the magMCM-CQ.

### 2.3. Characterization

The materials were characterized by the following techniques: (i) conventional powder X-ray diffraction (XRD6000, Shimadzu, Tokyo, Japan) using Cu-Kα radiation in low-angle range and in full range, using an internal standard (Si) when necessary. Commercial software (X’pert High Score, v. 1.0) was used for the reference intensity ratio (RIR) quantification of the phases identified in the diffractograms. (ii) Mössbauer spectroscopy (Wissel GmbH, Mömbris-Hohl, Germany) was employed to identify the iron oxide phases. Mössbauer spectra from (powder) samples were acquired in constant acceleration mode using a ^57^Co (Rh-matrix) source. The spectra, collected in transmission geometry at room temperature, were analyzed by a least square fitting method for the discrete Lorentzian lines at each hyperfine site using the software WinNormos (v. 3.0) for IgorPro (v. 6.1.2.1). The hyperfine parameters that identify the phases were taken from the literature. The isomer shift was stated relative to metallic iron. (iii) Material magnetization was measured using a MicroSense EZ9 vibrating sample magnetometer. (iv) N_2_ adsorption and desorption isotherms were obtained (Micromeritics Tristar II Krypton 3020, Norcross, GA, USA) and used to evaluate the textural properties of the materials. Surface area was determined using the Brunauer-Emmett-Teller (BET) method. Pore size distributions were assessed through the Barret-Joyner-Halenda (BJH) methodology and through density functional theory (DFT). (v) Scanning electron microscopy (EVO 50, Zeiss, Oberkochen, Germany) and transmission electron microscopy (JEM-1011, JEOL, Tokyo, Japan) were employed to evaluate the materials’ morphology. (vi) Thermal parameters were obtained with thermogravimetric analysis (TGA-50, Shimadzu, Tokyo, Japan) with a heating rate of 10 °C min^−1^ under air atmosphere. (vii) UV-Vis measurements were made using a spectrophotometer (Varian CARY 50 Conc UV-VIS, Agilent, Santa Clara, CA, USA). (viii) Differential scanning calorimetry (DSC) analyses (DSC-60, Shimadzu, Tokyo, Japan) were performed at a heating rate of 10 °C min^−1^, from room temperature to 250 °C, under a N_2_ flow of 50 mL min^−1^.

### 2.4. Dissolution/Release Assays

For the controlled release test, previously dried magMCM-CQ (12 mg) was dispersed in PBS buffer (pH 7.4, 80 mL) and kept under mechanical agitation. At specified time intervals, the material was separated magnetically, and 2 mL of the supernatant was collected for analysis. The same volume of fresh PBS was replaced to maintain sink conditions. This procedure was made in triplicate. For comparison purposes, crystalline CQ was kept in the same amount of PBS and agitation speed (*n* = 3) and, at specified time intervals, the solution was filtered and its concentration was evaluated. CQ concentration analysis was performed using a UV-Vis spectroscopy calibration curve (λ = 343 nm) in the range of 5 µg/mL to 33 µg/mL with a determination coefficient (R^2^) of 0.999. The kinetic profile for drug release was analyzed using the DDSolver add-in for Microsoft Excel 2016 [30] in which the data were fitted to zero order, first order, and Higuchi mathematical models.

### 2.5. Cell Culture and MTT Viability Test

The human non-tumoral cell line MRC-5 was purchased from the American Type Culture Collection (ATCC CCL-171, Manassas, VA, USA) and cultured in Dulbecco modified Eagle’s medium (DMEM, Vitrocell, Campinas, SP, Brazil) supplemented with 10% fetal bovine serum (FBS, Vitrocell, Campinas, SP, Brazil), 25 μg/mL amphotericin, and 50 μg/mL gentamicin, and maintained at 37 °C in a humidified atmosphere with 5% CO_2_. For cytotoxicity assessment, cells were seeded at a density of 1.5 × 10^4^ cells per well in 96-well plates and allowed to adhere for 24 h. The cell culture medium was removed, and the CQ, magMCM, and magMCM-CQ suspensions, recently prepared in cell culture medium with 0.5% of DMSO, were incubated with the attached cells at different concentrations (CQ: 0.00187, 0.0187, 0.187, 1.87, 18,7, 37.5, 75, and 150 μg/mL; magMCM: 0.0106, 0.106, 1.06, 10.6, 106, 212, 425, and 850 μg/mL; magMCM-CQ: 0.0125, 0.125, 1.25, 12.50, 125, 250, 500, and 1000 μg/mL). After 24 h of incubation, the cell culture medium was removed, the plate was washed twice with PBS and 50 μL of a 1 mg/mL solution of 3-(4,5-dimethylthiazol-2-yl)-2,5-diphenyltetrazolium bromide (MTT, Sigma-Aldrich, Burlington, MA, USA) was added to each well for 4 h at 37 °C. The PBS was removed, and the purple formazan crystals formed in the live cells were dissolved with DMSO (Vetec, Duque de Caxias, RJ, Brazil). Cell viability was determined by measuring the conversion of MTT to formazan using an automated microplate reader at 540 nm (SpectraMax M4, Molecular Device, San Jose, CA, USA). The percentage of cell viability was calculated relative to the control, and IC_50_ values (the concentration at which 50% of cells remain viable) were determined from the dose–response curves by plotting cell survival (%) against drug concentration (μg/mL) and calculated using the Quest Graph™ IC_50_ calculator (AAT Bioquest, Pleasanton, CA, USA), with errors estimated from triplicate experiments conducted independently three times.

## 3. Results and Discussion

**Figure 1:**
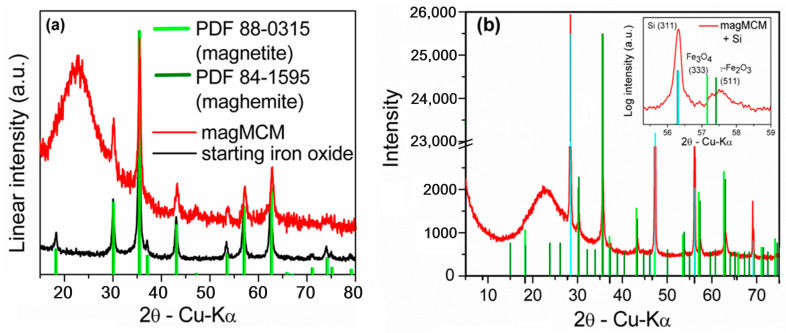
X-ray diffraction patterns of (**a**) starting iron oxide and magMCM samples; and (**b**) diffractogram of the magMCM sample with an internal standard (Si, identified by cyan bars). The inset in (**b**) was Cu Kα_2_ stripped and shows a detailed view of the range between 55.5° and 59°, where magnetite (333) and maghemite (511) present a perceptible difference and the Si (311) peak certifies that no 2-theta shift is present.

The crystal structure of the as-synthesized magnetic material (starting iron oxide) and after MCM-41 growth (magMCM) was assessed through X-ray diffraction and the obtained patterns are shown in Figure 1a, along with the standard peaks of the magnetite (PDF 85-0315) and maghemite (PDF 84-1595).

As can be observed in Figure 1a, the starting iron oxide sample exhibits peaks that are compatible with the interplanar distances of the face-centered cubic structure of magnetite (Fe_3_O_4_ PDF 88-0315) and maghemite (ɣ-Fe_2_O_3_ PDF 84-1595). Using the Scherrer method [31] with the full width at half maximum of the main peak (311 plane at 2θ = 35.4°), a grain size of 30 nm was estimated. Using the RIR (reference intensity ratio) method for an approximate quantification, the starting iron oxide presented around 60 wt.% magnetite and 40 wt.% maghemite. According to [32], an uncertainty of 3 wt.% is a reasonable estimate of the maximum uncertainty at the 95% confidence level for all phases analyzed. After MCM-41 silica formation in the magMCM sample, the characteristic amorphous silica halo from 2θ = 15° to 30° is observed. The crystalline reflections of the iron oxides are still visible, however, with less intensity due to the smaller content in the total sample. It can be seen that the peaks are slightly shifted to smaller interplanar distances at higher angles. The addition of an internal standard (silicon) allows a precise discrimination of the magnetite (333) and maghemite (511) peaks [33] between 57° and 58° (Figure 1b). The presence of the Si (311) peak certifies that no 2-theta shift is masking the small difference between the magnetite and the maghemite diffractograms.

Therefore, after the joint synthesis of the mesoporous silica with the embedded iron oxides, XRD reveals an increase in maghemite content, showing that the oxidation was only partially hindered by the addition of MCM-41. Additional experiments (Supplementary Information Figure S1) showed that the unprotected iron oxides treated at the same temperature (550 °C) and for the same time-lapse (6 h) in air were completely transformed to hematite.

**Figure 2:**
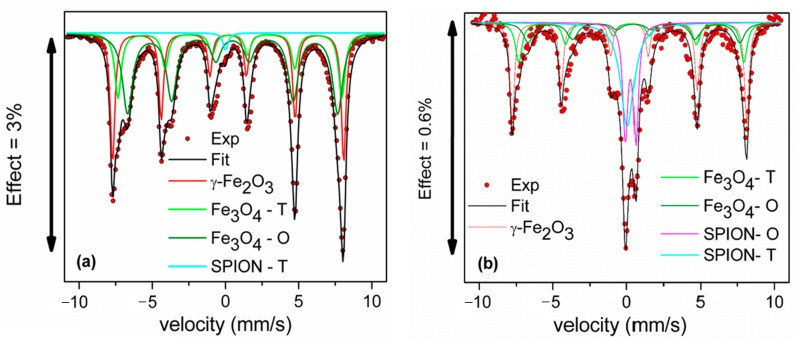
^57^Fe Mössbauer spectrum of (**a**) starting iron oxide and (**b**) magMCM sample.

Additional information on the iron oxides and magMCM can be obtained with ^57^Fe Mössbauer spectroscopy. The spectrum of the iron oxides (Figure 2a) was fitted with hyperfine parameters from the literature [34]. The dark green lines (two superposed indistinguishable sextets) are associated with maghemite, while magnetite presents two sextets (A tetrahedral and B octahedral sites), shown in light green. When the sums of the peak areas corresponding to a specific phase are compared in the starting iron oxide sample, magnetite is more abundant than maghemite. The magMCM spectrum (Figure 2b), fitted with the same parameters, showed a reduction of magnetite in relation to maghemite, as was observed in the XRD RIR analysis as well.

The most striking feature of the Mössbauer results, however, is the presence of asymmetric central doublets in the spectra, which are attributed to a superparamagnetic contribution of magnetite/maghemite nanoparticles. Superparamagnetism can occur when the grain size is so small that only single magnetic domains are supported in each grain [35] and superparamagnetic iron oxide nanoparticles are well known in the literature under the acronym SPION [36]. The subspectra attributed to SPIONs were fitted according to a previously reported approach [37,38] interpreting the magenta subspectrum (Figure 2a,b) as a size-induced paramagnetic doublet due to Fe atoms in tetrahedral sites (SPION-T) and the cyan subspectrum as a size-induced paramagnetic doublet due to Fe atoms in octahedral sites (SPION-O). It has to be noted that octahedral and tetrahedral sites are present in magnetite and maghemite structures; therefore, this analysis can conclude only the presence of nanoparticles, but not on the fraction of nanoparticles of each of these phases. The presence of SPIONs is small in the starting iron oxide, but very expressive in the magMCM sample. From the Mössbauer spectra, it can be concluded that magMCM contains a significant fraction (around 40 wt.%) of magnetite/maghemite nanoparticles, with dimensions below 10 nm, typical sizes at which nanoparticles display superparamagnetic behavior. The higher amount of SPIONs in the magMCM sample is probably due to the concentration of superparamagnetic particles during the magnetic recovery step. The SPIONs are strongly magnetized and will be sampled preferentially.

In order to assess the magnetic properties of the materials, magnetization hysteresis as a function of the applied field was obtained (Supplementary Information Figure S3). The hysteresis loops were classified as major loop type [39] and the saturation magnetization values were 57 emu g^−1^ and 12 emu g^−1^ for starting iron oxide and magMCM, respectively. The saturation magnetization value decreases as the proportion of magnetic material in the sample is reduced due to the addition of mesoporous silica. Even more, the presence of the maghemite can be responsible, to some extent, for the decrease in the magnetization, as the saturation magnetization of bulk maghemite is 74–80 emu g^−1^, while for bulk magnetite it is 84 emu g^−1^ [40]. Nevertheless, even with this phase modification, the materials still demonstrate appreciable magnetization (12 emu g^−1^), enabling their magnetic manipulation and separation.

**Figure 3:**
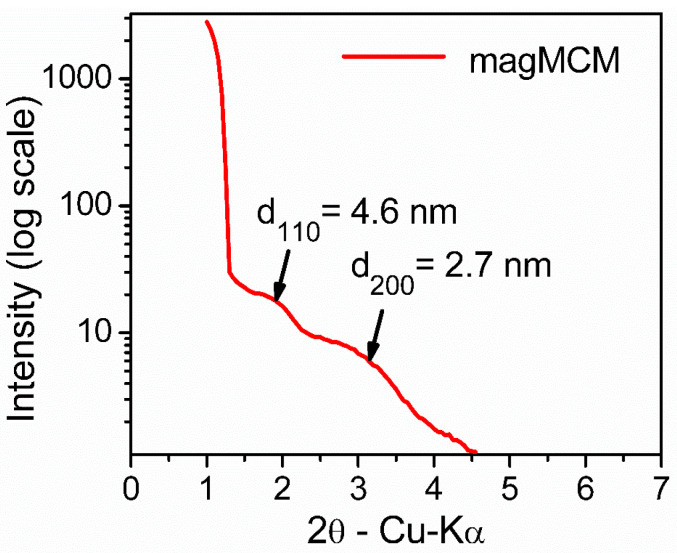
Low-angle X-ray diffraction pattern of the magMCM sample.

Regarding the mesoporous silica moiety in the magMCM sample, low-angle X-ray diffraction was used to characterize the arrangement of the mesopores. In Figure 3, the two characteristic peaks of the hexagonal pore arrangement (*p6mm*) of MCM-41 are observed; however, they shifted to lower angles than usually reported. The position of the 110 peak (interplanar distance of 4.6 nm) and the 200 peak (interplanar distance of 2.7 nm) indicate a channel repeat distance of around 5.4 nm. It has to be noted that this distance does not indicate the channel inner diameter, but the diameter plus silica walls. The broadening of the peaks indicates that the arrangement is not as regular as in pure MCM-41 [28], probably due to the decrease in long-range order introduced by the presence of magnetic particles during synthesis.

**Figure 4:**
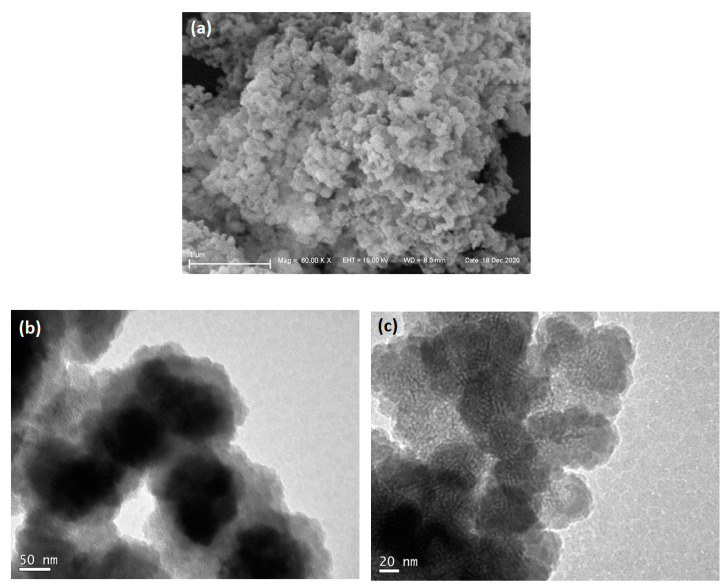
Electron microscopy images of magMCM: (**a**) SEM micrograph, (**b**) bright field TEM, and (**c**) high-resolution TEM images.

For morphological analysis, SEM and TEM images were obtained. In Figure 4a the SEM micrograph shows agglomerates of approximately spherical particles, 100 nm to 200 nm in diameter. In the TEM images (Figure 4b,c), it is possible to observe the particles individually. The high resolution of the channel voids in Figure 4c allows a direct measurement of the pore structure of the silica moiety. When grayscale profiles are taken perpendicularly to the channels in the image, the variation in brightness allows the determination of channel width to be around 4.9 nm (±0.5 nm) (Figure S2 Supplementary Materials). It can be seen that the long-range order is poor, and only half a dozen parallel channels can be found per ordered domain.

**Figure 5:**
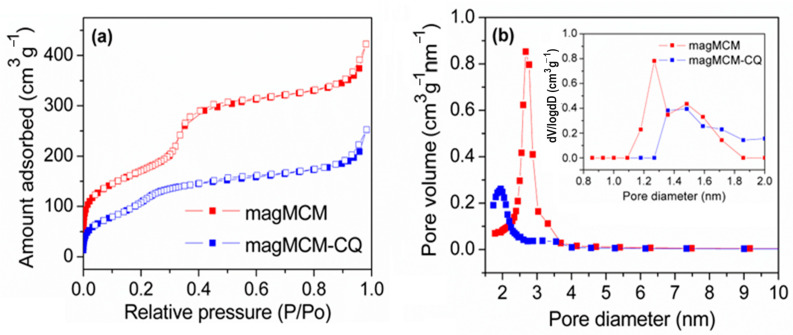
Textural analysis for magMCM and magMCM-CQ: (**a**) N_2_ adsorption and desorption isotherms; (**b**) pore size distribution by BJH and DFT (inset figure) methods.

The textural properties of the magMCM sample were studied by nitrogen adsorption and desorption isotherms. The isotherm and the pore size distribution (BJH and DFT methods) are depicted in Figure 5. The data from the textural analysis can be found in Table 1.

According to the data presented in Figure 5 and Table 1, magMCM exhibits a notable surface area and pore volume, presenting mesopores with 2.7 nm of diameter, compatible with the pore structure of non-magnetic MCM-41 and within the same order of magnitude as the observed channel diameter observed in the TEM image (Figure 4c). It is worth noting that in the literature, several attempts to synthesize magnetic MCM-41 materials have been reported, often resulting in a drastic surface area reduction that is not observed here [41,42,43]. The high surface area and the high pore volume of magMCM make it suitable to act as a magnetically responsive host of bioactive molecules such as drugs. After the encapsulation of CQ into the pores of magMCM, the textural analysis of magMCM-CQ presents a decrease in surface area, pore volume, and a shift in the pore size to 1.9 nm. These features suggest that chloroquine is filling the pores partially. The DFT micropore size distribution (inset Figure 5b) shows the same trend, with a decrease in microporosity after CQ encapsulation.

**Figure 6:**
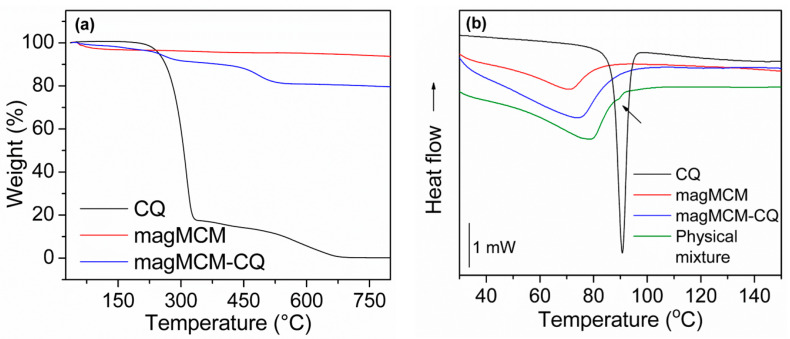
Thermal characterization of crystalline CQ, magMCM, and magMCM-CQ: (**a**) TGA and (**b**) DSC analyses.

The encapsulation of CQ in magMCM was performed in triplicate, yielding a drug loading of 14.9%. The TGA results (Figure 6a) show a drug content for one batch of 15.4%, which presents a good agreement with the experimentally designed value. Water desorption is responsible for the weight loss in magMCM and magMCM-CQ in the range of 0 °C to 150 °C [44]. The second weight loss in the range of 150 °C to 600 °C is attributed mainly to the CQ decomposition; however, in this temperature range, the dehydroxylation and condensation reactions of silanol groups also occur [45].

DSC analyses were performed to evaluate the CQ solid state in magMCM-CQ sample and the curves are presented in Figure 6b. The crystalline CQ melting point can be well observed as an endothermic peak at 90 °C [46]. The magMCM sample shows only a broad endothermic signal between 60 °C and 80 °C, which is related to the heat of vaporization of adsorbed water and appears for all the samples that contain silica. For magMCM-CQ, the absence of the endothermic peak of crystalline CQ seems to indicate that the drug underwent an amorphization process when encapsulated and dispersed at the magMCM surface. This hypothesis was supported by comparing the magMCM-CQ curve with its respective physical mixture (magMCM mixed with crystalline CQ), in which a signal of the CQ melting point can be seen, although with less intensity due to the small CQ content in the total sample. The amorphization process has been reported for silica-based mesoporous materials [47,48] and was attributed to the confinement effect of the drug by the pore walls [21]; small and dispersed quantities of the drug could have their crystallization capacity hampered, thereby increasing drug solubility [19,49,50].

**Figure 7:**
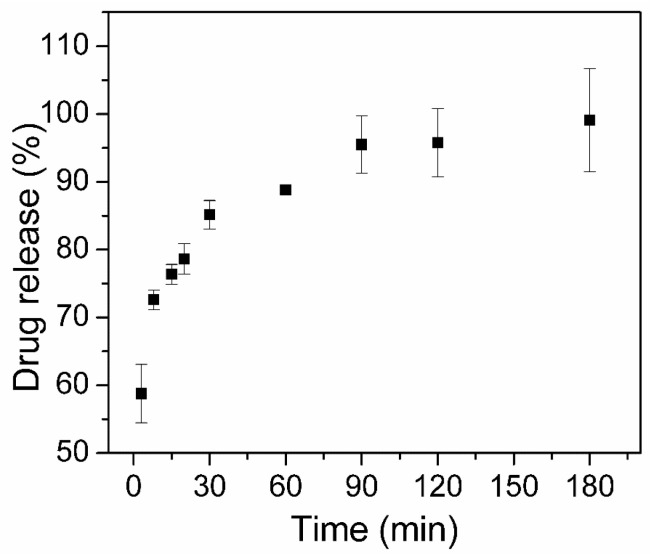
CQ in vitro drug dissolution and release from magMCM-CQ sample (*n* = 3).

One of the batches was used to evaluate the dissolution profile of encapsulated CQ in PBS at pH 7.4, and the obtained profile is presented in Figure 7. It is evident that there is a rapid dissolution of the CQ drug, with 99% released within 3 h. This observation is consistent with the textural data since there is a large available surface area and sufficient pore volume to facilitate the diffusion of the liquid medium within the pore channels, increasing the drug dissolution rate and carrying the solvated drug out of the nanocarrier. The best-fitted drug release mechanisms were the first-order model (R^2^ = 0.9468) where the drug release rate is concentration-dependent. This behavior is adequate for porous drug carriers with insolubility in water and was already reported for drug-loaded mesoporous silica, in which the high accessibility of the porous structure of silica enables unrestricted diffusion of the drug to the dissolution medium [51,52,53].

The dissolution of the non-encapsulated CQ in PBS was evaluated under the same conditions at 3, 90, and 180 min, and a comparison between the encapsulated (magMCM-CQ) and non-encapsulated CQ is summarized in Table 2. It can be observed that encapsulating the CQ drug within the material enhances the rate at which the drug dissolves in the medium. Only 66% of the non-encapsulated drug is dissolved in 90 min, whereas 95.5% of the encapsulated drug is dissolved in the same conditions. This result can be related to the amorphization process of the drug, which was observed by the DSC analysis.

Mesoporous silica nanoparticles have been extensively evaluated for their cytotoxicity and biocompatibility in both tumor and non-tumor cell lines. Studies have consistently shown that MCM-41 exhibits low cytotoxicity towards non-tumor cells, indicating its biocompatibility and potential for biomedical applications [54,55,56].

**Figure 8:**
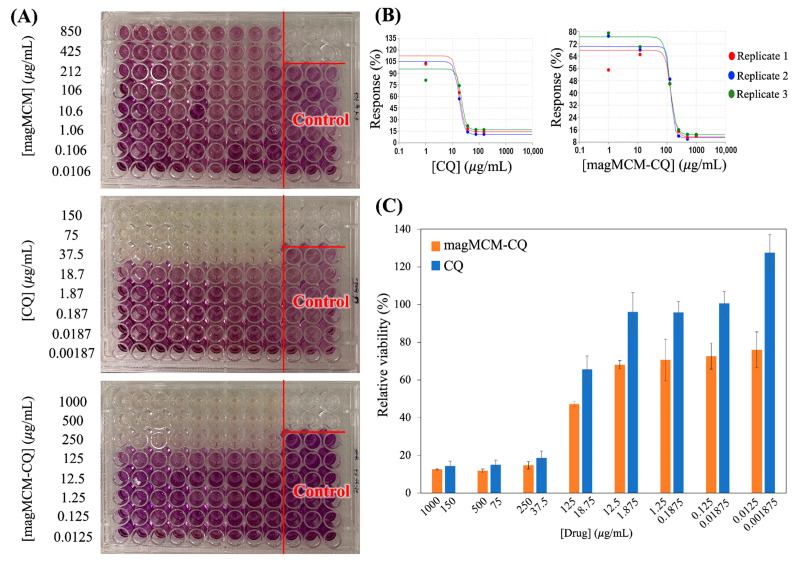
(**A**) MTT assays in human non-tumoral cell line MRC-5 in 96-well plates incubated with suspensions of magMCM, CQ, and magMCM-CQ for 24 h; (**B**) dose–response curve of treatment in MTT assays in human non-tumoral cell line MRC-5 incubated with CQ and magMCM-CQ for 24 h created by Quest Graph™ IC_50_ calculator; (**C**) the histogram of MTT assay comparing the proliferation of cell line MRC-5 in several concentrations of CQ and magMCM-CQ for 24 h. Error bars represent the standard deviation of three independent experiments.

The evaluation of cytotoxicity conducted on the non-tumoral MRC-5 cell line over a 24 h incubation period at eight different concentrations using the MTT assay with CQ, magMCM, and magMCM-CQ formulations provided valuable insights into their biocompatibility and therapeutic potential (Figure 8A). The results demonstrated that magMCM exhibited excellent biocompatibility, as evidenced by its lack of cytotoxicity up to the highest tested concentration of 850 μg/mL, as previously described. The IC_50_ values for non-encapsulated CQ and magMCM-CQ were determined as 20.53 ± 1.92 and 113.95 ± 4.76 μg/mL, respectively, showing that the encapsulation of CQ within magMCM resulted in a significant increase in the IC_50_ value compared to magMCM, indicating a potential for sustained drug release (Figure 8B,C). Comparative analysis of cytotoxicity revealed that the inhibitory activity occurred at equivalent doses, taking into account a ca. 15% CQ loading in the magMCM-CQ, as described above in the TGA results. The results presented herein align with findings from previous studies investigating similar systems. MSN functionalized with lactose for liver targeting and docetaxel delivery showed increased drug concentration at target sites and the MSN did not display a cytotoxic effect in the maximal concentration of 200 μg mL^−1^ [57]. Folic acid-modified MSNs targeting breast cancer cells presented enhanced drug delivery and therapeutic efficacy in target organs [58,59]. Moreover, MSN doped with Eu and Gd and functionalized with hyaluronic acid was tested against mouse fibroblasts (L929) and human lung adenocarcinoma cells (A549), showing that MSN material itself was not cytotoxic to the cells at 200 μg mL^−1^ [60].

Notably, all these studies highlight the biocompatibility of MSN, as evidenced by their negligible cytotoxic effects on various cell lines. These findings underscore the promising role of magMCM as a biocompatible carrier for drug delivery, the unchanged biological activity of the drug, and its potential applications in targeted therapy.

## 4. Conclusions

In this work, a mesoporous silica-based material with dispersed magnetic particles (magMCM) was obtained and tested as a drug delivery system with the antimalarial drug chloroquine (CQ). The magnetic phase of magMCM was a mixture of iron oxide phases, with the primary phase identified as γ-Fe_2_O_3_ (maghemite) and containing a significant quantity of superparamagnetic iron oxide nanoparticles. Magnetization hysteresis confirmed the material’s response to a magnetic field. In low-angle X-ray diffraction, characteristic peaks of the hexagonal MCM-41 pore arrangement were observed. The pore channels were observable in TEM images. Using the N_2_ adsorption–desorption isotherm, the specific surface area and pore diameter of magMCM were determined to be 630 m^2^ g^−1^ and 2.7 nm, respectively. After drug encapsulation, the specific surface area decreased to 467 m^2^ g^−1^, and the pore diameter reduced to 1.9 nm. A substantial decrease in porosity indicates that the CQ drug is allocated in the mesoporous channels, although it does not fill them completely. Finally, controlled release tests of the drug CQ showed a rapid dissolution and release profile (99% released within 3 h), which is expected due to the well-dispersed drug molecules on the pore walls, facilitating diffusion of the liquid medium into the pores to dissolve and release the drug from the nanocarrier. The increase in dissolution rate from the magMCM-CQ material in comparison with non-encapsulated CQ suggests that the encapsulation of CQ is in amorphous form. Therefore, the material magMCM has the potential to be applied as a magnetically controlled drug delivery system for poorly soluble drugs, such as antimalarials, thereby enhancing treatment efficiency, as displayed in the in vitro MTT assay against the non-tumoral MRC-5 cell line.

## Figures and Tables

**Table 1:** Textural data for magMCM and magMCM-CQ materials.

Sample	S_BET_ (±5 m^2^ g^−1^)	Pore Volume (±0.001 cm^3^ g^−1^)	Main Pore Size ^a^ (nm)
magMCM	624	0.649	2.7
magMCM-CQ	467	0.346	1.9

a = obtained from the maximum of the BJH pore distribution peak.

**Table 2:** Dissolution data of free CQ and released CQ in PBS at specified time points.

Time	Non-Encapsulated CQ (% CQ Released)	magMCM-CQ (% CQ Released)
3	17.3	58.8
90	65.9	95.5
180	94.1	99.1

## Data Availability

The raw data supporting the conclusions of this article will be made available by the authors upon request.

## Supplementary Materials

The following supporting information can be downloaded at: https://www.mdpi.com/article/10.3390/pharmaceutics16030357/s1, Figure S1: X-ray diffraction pattern of starting iron oxide sample after calcination (550 °C); Figure S2: Brightness intensity profile obtained on the yellow line in the high-resolution TEM image; Figure S3: Magnetization hysteresis of starting iron oxide and magMCM samples.
