# Supplementary material for: Magnetic Mesoporous Silica for Targeted Drug Delivery of Chloroquine: Synthesis, Characterization, and In Vitro Evaluation
Source: Pharmaceutics. 2024 Mar 3;16(3):357. doi: 10.3390/pharmaceutics16030357 (PMC10975348; doi:10.3390/pharmaceutics16030357)
Supplement: Supplementary file 1 [file pharmaceutics-16-00357-s001.zip › pharmaceutics-2874806-supplementary.pdf]

Magnetic mesoporous silica for targeted drug delivery of chloroquine: synthesis, characterization and *in vitro* evaluation

Rafaela de Andrade[1], Rita de Cássia dos Reis Schmidt[2], Leonardo Santos Gomes[1], Legna Colina-Vegas[1], Ruth Hinrichs[3], Marcos A.Z. Vasconcellos[4], Tania Maria Haas Costa[1], Monique Deon[2]*, Wilmer Villarreal[1], Edilson V. Benvenutti[1]*

[1] Instituto de Química, Universidade Federal do Rio Grande do Sul (UFRGS), PO Box 15003, 91501970, Porto Alegre, RS, Brazil
[2] Programa de Pós-Graduação em Biociências, Universidade Federal de Ciências da Saúde de Porto Alegre (UFCSPA), 90050170, Porto Alegre, RS, Brazil
[3] Instituto de Geociências, Universidade Federal do Rio Grande do Sul (UFRGS), PO Box 15001, 91501970, Porto Alegre, RS, Brazil
[4] Instituto de Física, Universidade Federal do Rio Grande do Sul (UFRGS), PO Box 15051, 91501970, Porto Alegre, RS, Brazil
* Correspondence: MD: monique.deon@ufcspa.edu.br, EB: benvenutti@ufrgs.br

Supplementary information

XRD of the starting iron oxides subjected to a calcination process equivalent to the calcination of magMCM, showing the complete transformation of magnetite/maghemite to hematite.

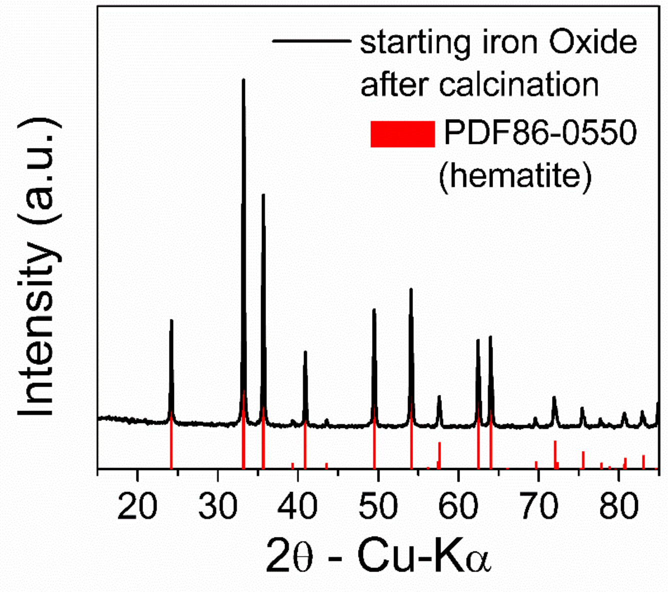

Figure S1. Starting iron oxides were submitted to thermal treatment (550 °C) in air for 6 h. The diffractogram shows complete transformation to hematite (α-Fe_2O_3).

In order to obtain the channel repeat distance directly from the TEM image, Fig. 4c was calibrated using the software Image J (Developer: National Institutes of Health; License: Public Domain, BSD-2). Brightness (grey scale) profiles (ctrl+K) were obtained on several lines, which were traced perpendicularly to channel direction, as exemplified with the yellow line in the TEM image of Fig. S2. On the calibrated profile,

the peak to peak distance can be determined using the command "measure" (ctrl+M). After the completion of sufficient measurements on several profiles, Image J offers the possibility to summarize the results and to present mean and standard deviation in the Results table.

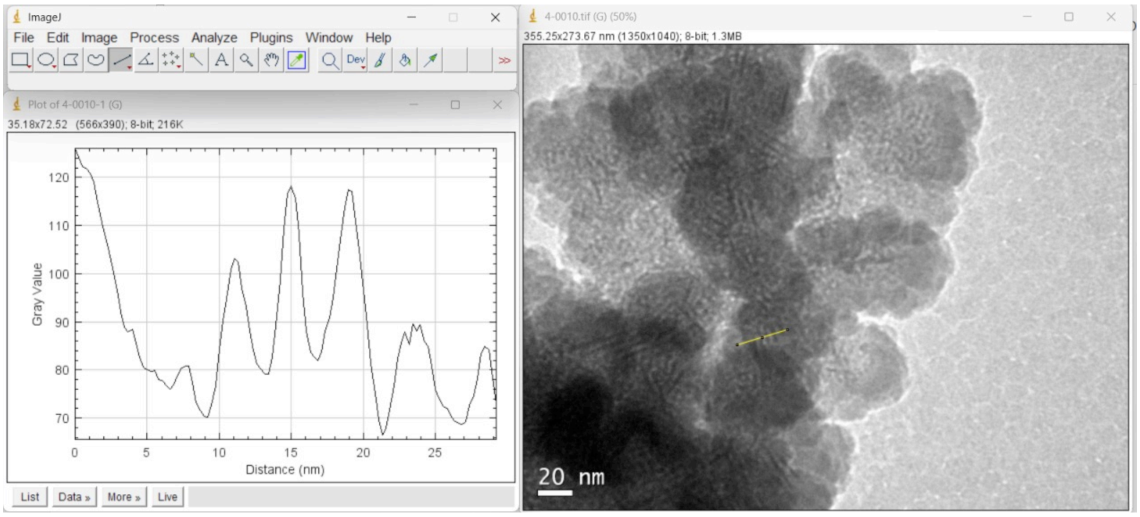

Figure S2. Brightness intensity profile obtained on the yellow line the in High Resolution TEM image.

Profiles were obtained in several locations, where the channels are parallel and the peaks could be clearly distinguished. Thirty measurements were performed, resulting in a mean of 4.9 nm (standard deviation of 0.5 nm) for the channel repeat distance.

It has to be kept in mind that TEM data are not as representative of the whole sample as e.g. XRD measurements, where the technique samples thousands of grains, instead of a dozen or so, as in TEM.

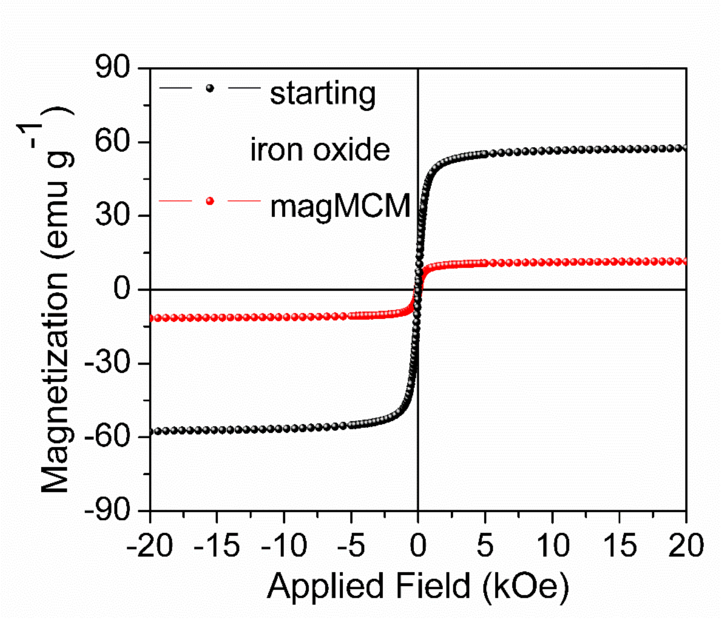

Figure S3. Magnetization hysteresis of starting iron oxide and magMCM samples.
